# Supplementary material for: Time preferences are reliable across time-horizons and verbal versus experiential tasks
Source: eLife. 2019 Feb 5;8:e39656. doi: 10.7554/eLife.39656 (PMC6363390; doi:10.7554/eLife.39656)
Supplement: Supplementary file 3. — Each plot is the softmax-hyperbolic fit and data for each subject in control experiment 2. In each panel, the marker and error bar indicate the mean and binomial confidence intervals of the subjects choices for that offer. The smooth ribbon indicated the BHM model fits (at 50, 80, 99% credible intervals). At the top of each subject plot we indicate the mean estimates of log⁡(k) and τ for each task for that subject. We also indicate the Bayesian r2 for each task. Plots from Left to right, row-by-row are ordered by discount factor for SV. [file elife-39656-supp3.pdf]

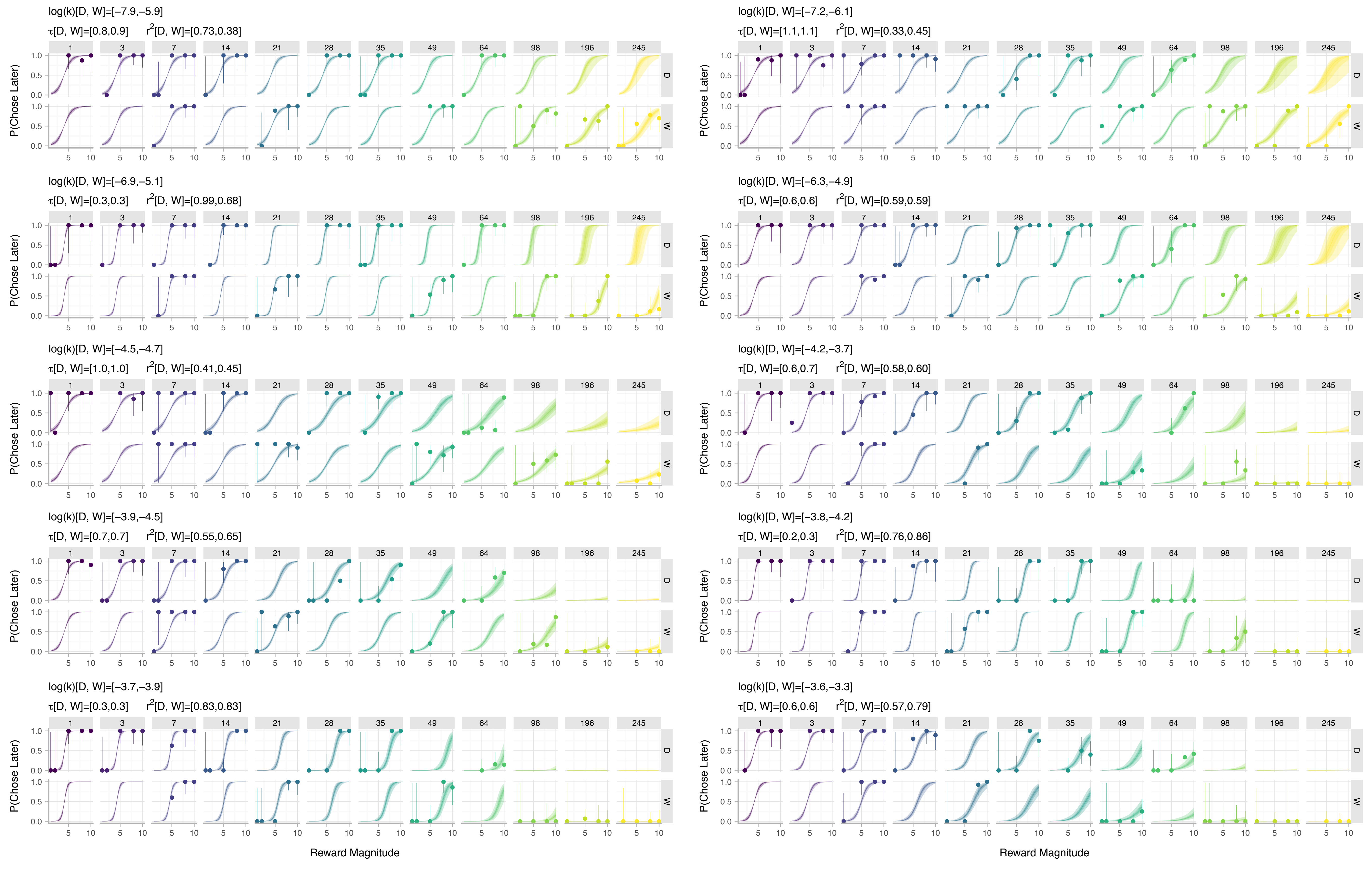

log(k)[D, W]=[−7.9,−5.9]
τ[D, W]=[0.8,0.9]
r²[D, W]=[0.73,0.38]
log(k)[D, W]=[−7.2,−6.1]
τ[D, W]=[1.1,1.1]
r²[D, W]=[0.33,0.45]
log(k)[D, W]=[−6.9,−5.1]
τ[D, W]=[0.3,0.3]
r²[D, W]=[0.99,0.68]
log(k)[D, W]=[−6.3,−4.9]
τ[D, W]=[0.6,0.6]
r²[D, W]=[0.59,0.59]
log(k)[D, W]=[−4.5,−4.7]
τ[D, W]=[1.0,1.0]
r²[D, W]=[0.41,0.45]
log(k)[D, W]=[−4.2,−3.7]
τ[D, W]=[0.6,0.7]
r²[D, W]=[0.58,0.60]
log(k)[D, W]=[−3.9,−4.5]
τ[D, W]=[0.7,0.7]
r²[D, W]=[0.55,0.65]
log(k)[D, W]=[−3.8,−4.2]
τ[D, W]=[0.2,0.3]
r²[D, W]=[0.76,0.86]
log(k)[D, W]=[−3.7,−3.9]
τ[D, W]=[0.3,0.3]
r²[D, W]=[0.83,0.83]
log(k)[D, W]=[−3.6,−3.3]
τ[D, W]=[0.6,0.6]
r²[D, W]=[0.57,0.79]
1 3 7 14 21 28 35 49 64 98 196 245
D
W
P(Chose Later)
Reward Magnitude

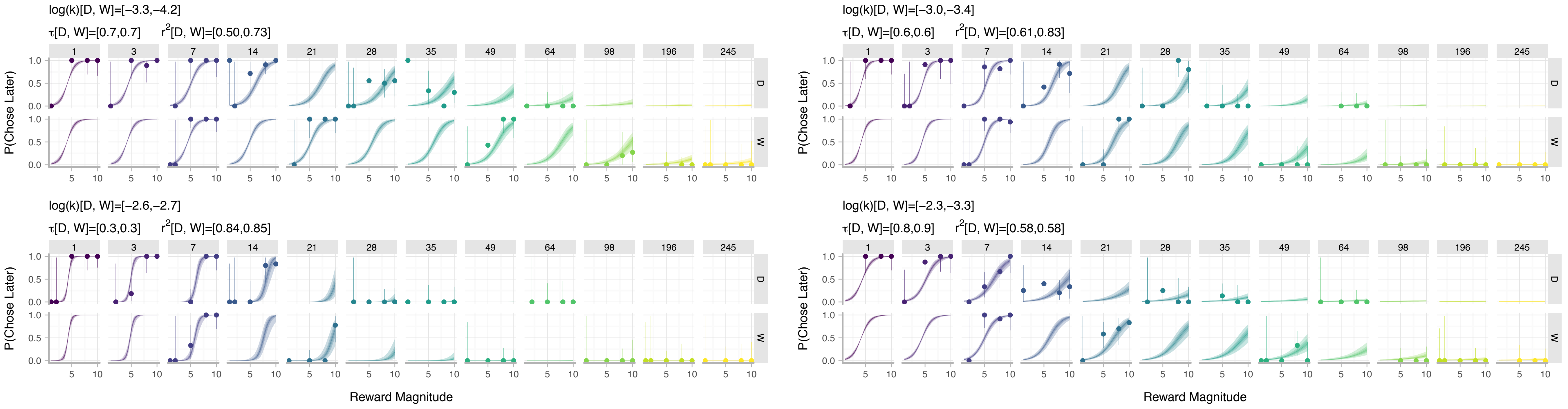

log(k)[D, W]=[−3.3,−4.2]
τ[D, W]=[0.7,0.7]
r^2[D, W]=[0.50,0.73]
log(k)[D, W]=[−3.0,−3.4]
τ[D, W]=[0.6,0.6]
r^2[D, W]=[0.61,0.83]
log(k)[D, W]=[−2.6,−2.7]
τ[D, W]=[0.3,0.3]
r^2[D, W]=[0.84,0.85]
log(k)[D, W]=[−2.3,−3.3]
τ[D, W]=[0.8,0.9]
r^2[D, W]=[0.58,0.58]
1 3 7 14 21 28 35 49 64 98 196 245
D
W
P(Chose Later)
0.0 0.5 1.0
5 10
Reward Magnitude
