# Supplementary material for: Time preferences are reliable across time-horizons and verbal versus experiential tasks
Source: eLife. 2019 Feb 5;8:e39656. doi: 10.7554/eLife.39656 (PMC6363390; doi:10.7554/eLife.39656)
Supplement: Supplementary file 4. [file elife-39656-supp4.pdf]

Experiment instructions

A. Nonverbal experiment:

Welcome Everyone,

The first order of business is to sign the informed consent form. If you have not already done that, could you raise your hand and we will get it to you. You should know that everyone here has been recruited in the same way. **Also, there is no deception involved in this experiment. Everything that we tell you is true. This is the standard practice in economics.**

You will be taking part in a study of individual preferences. We are running this study in parallel with an animal study, so you will not receive any explicit instructions using language about what you will see or what you will do. The only thing we can say now is **USE THE MOUSE** and react to different visual stimuli that appear on the screen and sound stimuli that you hear. We would like you to play the game and earn coins. You need to figure out the rules of the game by going through the learning stages on your own. Once you pass the learning stages you will proceed with the game based on the rules you learned during the learning stages.

There are two stages to the experiment: learning stages and then the decision stages.

You will earn ___ RMB just for participation. The coins you earn in the learning stages are not converted to RMB. However, completing learning stages is essential to advance to the decision stages. Coins you earn in the decision stages will be converted to RMB at the following rate: **1 coin = ___ RMB**.

Any questions??

If there are no further questions, please sign the consent form and fill out the survey. When we begin the game, we will give you time to adjust sound volume, so it is comfortable for you. During the learning stages and the game when the program tells you to report to the experimenter please silently raise your hand.

In the game, do not rush. Take your time to make decisions. After the experiment is finished, please fill out the feedback survey. Thank you!

You are making an important scientific contribution, but due to the nature of the experiments, we will only be able to explain the scientific questions once you have fully completed your participation. We also ask you not to discuss the task details with other students, since if they decide to participate in the future it may influence the results. If you do want to say something just tell them you are participating in an experiment, and if they want to know more they should participate as well. :)
