# Supplementary material for: Time preferences are reliable across time-horizons and verbal versus experiential tasks
Source: eLife. 2019 Feb 5;8:e39656. doi: 10.7554/eLife.39656 (PMC6363390; doi:10.7554/eLife.39656)
Supplement: Supplementary file 5. [file elife-39656-supp5.pdf]

Experiment instructions

B. Verbal experiment:

Welcome Everyone,

The first order of business is to sign the informed consent form. If you have not already done that, could you raise your hand and we will get it to you. You should know that everyone here has been recruited in the same way. **Also, there is no deception involved in this experiment. Everything that we tell you is true. This is the standard practice in economics.**

We would like you to play the game and earn coins. In each trial of this game you will be asked to make a choice between getting some number of coins now and getting some number of coins after a certain delay. There are four stages to the experiment: 2 **short** delays and 2 **long** delays stages.

During the short delays stage, you will wait for the delay associated with your choice before receiving the indicated number of coins. Then you will go to the next trial. For example, if you choose to wait for 14 seconds for 8 coins, then you will wait 14 seconds before receiving the coins and then you will proceed to the next trial. At the end of the session the coins you earn in this stage will be converted into RMB and you will be paid (either in cash, wechat or alipay). **All coins you earned in this stage will be counted for the payment.**

In the long delays stage, the choices you make will be recorded but you will not wait before proceeding to the next trial. At the end of the experiment **one trial** from 2 long delay stages will be selected randomly and your choice on that trial will be fulfilled. For example, if on the randomly selected trial you choose "10 Coins in 3 days", then in 3 days we will transfer (or pay) the RMB equivalent of 10 coins. If on the randomly selected trial you chose "4 coins today" you will receive the RMB equivalent of 4 coins at the end of the session today.

You will earn ___ RMB just for participation. Coins you earn in the 2 **short** delay stages will be converted to RMB at the following rate: **1 coin = ___ RMB**. Coins you earn on the randomly selected trial from the long delay stages will be converted to RMB at the following rate: **1 coin = ___ RMB**. In terms of money you can earn short delays stages are equal on average to long delays stages.

Any questions??

If there are no further questions, please sign the consent form and fill out the survey. When we begin the game, we will give you time to adjust sound volume, so it is comfortable for you. In the game, do not rush. Take your time to make decisions. After the experiment is finished, please fill out the feedback survey. Thank you!

You are making an important scientific contribution, but due to the nature of the experiments, we will only be able to explain the scientific questions once you have fully completed your participation. We also ask you not to discuss the task details with other students, since if they decide to participate in the future it may influence the results. If you do want to say something just tell them you are participating in an experiment, and if they want to know more they should participate as well. :)

You will receive _____ RMB ___________

Today's date, time __________

Experimenter's signature ___________
