# Supplementary material for: Time preferences are reliable across time-horizons and verbal versus experiential tasks
Source: eLife. 2019 Feb 5;8:e39656. doi: 10.7554/eLife.39656 (PMC6363390; doi:10.7554/eLife.39656)
Supplement: Transparent reporting form [file elife-39656-transrepform.docx]

***eLife’s* transparent reporting form**

We encourage authors to provide detailed information *within their submission* to facilitate the interpretation and replication of experiments. Authors can upload supporting documentation to indicate the use of appropriate reporting guidelines for health-related research (see [EQUATOR Network](http://www.equator-network.org/%20)), life science research (see the [BioSharing Information Resource](https://biosharing.org/)), or the [ARRIVE guidelines](http://www.plosbiology.org/article/info:doi/10.1371/journal.pbio.1000412) for reporting work involving animal research. Where applicable, authors should refer to any relevant reporting standards documents in this form.

If you have any questions, please consult our Journal Policies and/or contact us: [editorial@elifesciences.org](mailto:editorial@elifesciences.org).

**Sample-size estimation**

- You should state whether an appropriate sample size was computed when the study was being designed
- You should state the statistical method of sample size computation and any required assumptions
- If no explicit power analysis was used, you should describe how you decided what sample (replicate) size (number) to use

Please outline where this information can be found within the submission (e.g., sections or figure legends), or explain why this information doesn’t apply to your submission:

We initially started with 35 subjects for the main experiment and doubled the sample size due to power calculations (Cohen, 1988; Bonett et al., 2000) described in Materials and Methods / Participants.

**Replicates**

- You should report how often each experiment was performed
- You should include a definition of biological versus technical replication
- The data obtained should be provided and sufficient information should be provided to indicate the number of independent biological and/or technical replicates
- If you encountered any outliers, you should describe how these were handled
- Criteria for exclusion/inclusion of data should be clearly stated
- High-throughput sequence data should be uploaded before submission, with a private link for reviewers provided (these are available from both GEO and ArrayExpress)

Please outline where this information can be found within the submission (e.g., sections or figure legends), or explain why this information doesn’t apply to your submission:

The number of repetitions of each task in the experiments is reported in Materials and Methods / Participants; Materials and Methods/ Experimental design. The excluded subject data due to withdrawal was destroyed as per approved IRB protocol. The excluded session data due to first-order violations is reported in Materials and Methods/ Analysis.
Data is provided via a github repository with a repository DOI provided by zenodo.

**Statistical reporting**

- Statistical analysis methods should be described and justified
- Raw data should be presented in figures whenever informative to do so (typically when N per group is less than 10)
- For each experiment, you should identify the statistical tests used, exact values of N, definitions of center, methods of multiple test correction, and dispersion and precision measures (e.g., mean, median, SD, SEM, confidence intervals; and, for the major substantive results, a measure of effect size (e.g., Pearson's r, Cohen's d)
- Report exact p-values wherever possible alongside the summary statistics and 95% confidence intervals. These should be reported for all key questions and not only when the p-value is less than 0.05.

Please outline where this information can be found within the submission (e.g., sections or figure legends), or explain why this information doesn’t apply to your submission:

Types of statistical analysis, test results and significance levels are presented in Figures 2-5 (including supplemental figures), all Tables, Results, Materials and Methods/Analysis sections. We have not included exact p values for all tests to facilitate flow of reading, but we can do so if necessary (or add additional figure supplements).

(For large datasets, or papers with a very large number of statistical tests, you may upload a single table file with tests, Ns, etc., with reference to sections in the manuscript.)

**Group allocation**

- Indicate how samples were allocated into experimental groups (in the case of clinical studies, please specify allocation to treatment method); if randomization was used, please also state if restricted randomization was applied
- Indicate if masking was used during group allocation, data collection and/or data analysis

Please outline where this information can be found within the submission (e.g., sections or figure legends), or explain why this information doesn’t apply to your submission:

Our main experiment uses a within-subject design. After analysis of the main experimental data, we recruited more subjects for two control experiments to aid in the interpretation of the main experimental results.

**Additional data files (“source data”)**

- We encourage you to upload relevant additional data files, such as numerical data that are represented as a graph in a figure, or as a summary table
- Where provided, these should be in the most useful format, and they can be uploaded as “Source data” files linked to a main figure or table
- Include model definition files including the full list of parameters used
- Include code used for data analysis (e.g., R, MatLab)
- Avoid stating that data files are “available upon request”

Please indicate the figures or tables for which source data files have been provided:

Software for running the task, as well as the data and analysis code for regenerating our results are available at github repository. The code that generates all main figures (2-5) in Matlab is provided. The R code (and data) for fitten the hierarchical models is also included in the github repository.

This is described in Materials and Methods/Software, Materials and Methods/Data Availability
